# Human papillomavirus prevalence and vaccine effectiveness in young women in Germany, 2017/2018: results from a nationwide study

**DOI:** 10.3389/fpubh.2023.1204101

**Published:** 2023-08-31

**Authors:** Anna Loenenbach, Viktoria Schönfeld, Anja Takla, Miriam Wiese-Posselt, Adine Marquis, Sarah Thies, Matthias Sand, Andreas M. Kaufmann, Ole Wichmann, Thomas Harder

**Affiliations:** ^1^Immunization Unit, Robert Koch-Institute, Berlin, Germany; ^2^Institute of Hygiene and Environmental Medicine, Charité—Universitätsmedizin Berlin Corporate Member of Freie Universität Berlin, Humboldt-Universität zu Berlin, Berlin, Germany; ^3^Department of Gynecology, HPV Research Laboratory, Charité—Universitätsmedizin Berlin, Corporate Member of Freie Universität Berlin and Humboldt-Universität zu Berlin, Berlin, Germany; ^4^GESIS Leibniz Institute for Social Sciences, Mannheim, Germany

**Keywords:** population-based study, cervical cancer prevention, self-sampling, HPV genotyping, vaccine effectiveness, prevalence

## Abstract

**Background:**

Infections with human papillomaviruses (HPV) are sexually transmitted and can cause cancer. In Germany, vaccination against HPV is recommended for girls and boys aged 9–17 years. We aimed to investigate HPV DNA prevalence, genotype distribution and vaccine effectiveness (VE) in women aged 20–25 years 10 years after the introduction of HPV vaccination in Germany (2018–2019), and compared these data to an equally designed study from 2010–2012.

**Methods:**

Seventy six geographical clusters were randomly selected, followed by random selection of 61 women aged 20–25 years per cluster. Participants performed cervicovaginal self-sampling and answered questions on demographics, sexual behaviour and HPV vaccination. Samples were tested for 18 high risk and nine low risk HPV genotypes. We performed chi-square tests, Fisher’s exact test, unpaired Student’s *t*-test and proportion *t*-test, and calculated crude and adjusted prevalence ratios (PR) and 95% CIs.

**Results:**

Of 7,858 contacted women a total of 1,226 agreed to participate. Of these, 94 women were positive for HPV types 16 and/or 18. HPV16 prevalence was 7.0% (95% CI 5.6–8.6) and HPV18 prevalence was 0.8% (95% CI 0.4–1.5). HPV6 and HPV11 were rare with only five (0.4%; 0.1–0.9) and one (0%; 95% CI 0.0–0.5) positive tests. Seven hundred fifty-seven women (62%) had received at least one HPV vaccine dose and 348 (28%) were vaccinated as currently recommended. Confounder-adjusted VE was 46.4% (95% CI 4.2–70.1) against HPV16/18 infection and 49.1% (95% CI 8.2–71.8) against infection with at least one HPV genotype covered by the quadrivalent HPV vaccine. Compared with the 2010–2012 study results, HPV16/18 prevalence dropped from 22.5% (95% CI 19.0–26.3) to 10.3% (95% CI 7.5–13.9; *p* < 0.0001) in unvaccinated participants.

**Conclusion:**

Vaccine-covered HPV genotypes were rare among 20–25 years old women in Germany and decreased compared to the time point shortly after the start of the HPV vaccination program. HPV prevalence of almost all vaccine-covered genotypes was strongly reduced in vaccinated participants. A decrease of HPV16 and HPV18 was even observed in unvaccinated participants, compared to 2010–2012 data, suggesting indirect protection of unvaccinated women. Low VE against HPV16/18 and HPV6/11/16/18 in our study might be attributable to study design in combination with the endpoint selection of (mainly transient) HPV DNA positivity.

## Introduction

Infections with human papillomaviruses (HPV) can cause dysplasia and cancer (1). They are among the most common sexually transmitted infections worldwide (2, 3). Mucosal HPV infecting the anogenital area is highly transmissible, and sexually active persons become usually infected during their first sexual contacts (1). Most HPV infections do not persist, resolve spontaneously and are no longer detectable within 2 years after infection (1, 4). However, in about 10% of HPV-infected persons, the virus persists and can lead to precancerous lesions and invasive cancer (1). The majority of cervical cancer is caused by HPV types 16 and 18, with type HPV16 being the most oncogenic type (5). Apart from the development of cervical cancer, HPV infections are also associated with other anogenital dysplasia and cancers at vaginal, vulvar, anal, penile as well as head and neck regions. There are approximately 40 different HPV alpha genotypes, that infect the anogenital mucosa, which are classified according to their oncogenic potential into high-risk (HR)-HPV (genotypes 16, 18, 31, 33, 35, 39, 45, 51, 52, 56, 58, 59), probable HR-HPV (genotypes 66, 68), potential HR-HPV (genotypes 26, 53, 73, 82), and low-risk (LR) types HPV6 and HPV11 and others (6). Some of the LR-HPV genotypes are responsible for the development of genital warts and only rarely induce invasive cancers.

The HPV prevalence differs according to HPV genotype and highly depends on the site of sampling and test method (7, 8). In a cross-sectional study utilizing a self-sampling cervicovaginal lavage method approximately 20% of 20 to 25 years-old women in Germany were HPV16 positive, while about 5% were positive for HPV18 (9). Further studies from Germany reported a HR-HPV prevalence of 23% to 28% for women younger than 30 years (7, 10). In all these studies, the majority of participating women had not been vaccinated against HPV.

Effective prophylactic vaccines against HPV are available since 2006 and since 2016 the nine-valent vaccine has been mainly used in Germany (11, 12). Currently, the German Standing Committee on Vaccination (Ständige Impfkommission, STIKO) recommends vaccination against HPV for all girls and boys aged 9–14 years and catch-up vaccination up to the age of 17 years (13). For complete immunization, STIKO recommends two doses at least 5 months apart for the age group 9–14 years, and three doses for adolescents aged 15 years and older. In Germany, vaccination against HPV is free of charge for the recommended age group. However, vaccine uptake is still low with 51% of the 15 years old girls and 17% of the 15 years old boys having received a full vaccination course in 2020 (14).

To determine the baseline HPV prevalence, genotype distribution and risk factors for HPV-infection in 20–25 years old women in Germany, the Robert Koch Institute (RKI) and the HPV laboratory at the Clinic for Gynecology of Charité—Universitätsmedizin Berlin conducted an HPV prevalence study (baseline study) in the years 2010–2012 (15). This study targeted birth cohorts that were eligible for HPV-vaccination shortly after roll-out of the routine HPV immunization program. Among 787 included women, 512 (65%) had not been vaccinated against HPV. In the non-vaccinated population HPV16 was with 20% the most prevalent HPV type.

The present study aimed to repeat the 2010–2012 baseline study with a similar study design in 2017/2018 (i.e., 10 years after initiation of routine HPV immunization) by collecting data on HPV infections in a nation-wide sample of 20–25 years-old women in Germany, which allows direct comparison between these two time points. We investigated the prevalence and vaccine effectiveness (VE), and compared the results to the baseline study in order to contribute to the evaluation of the vaccination program in Germany.

## Materials and methods

### Study population and recruitment

This population-based cross-sectional study was carried out by the RKI in collaboration with the Clinic for Gynecology, HPV research lab at Charité—Universitätsmedizin Berlin. The recruitment strategy for the population-based random sample was developed by GESIS Leibniz Institute for Social Sciences (Mannheim/Cologne, Germany). Recruitment of participants was carried out from June 2017 to January 2018 using a two-stage sample design. Seventy six geographical clusters (at community level/municipalities) were randomly selected, followed by the random selection of 61 women aged 20 to 25 years in each of these clusters. Clusters were formed according to region (eastern and western federal states) and number of inhabitants (rural <100,000 or urban >100,000 inhabitants per community). An additional cluster was selected in the city of Berlin with 400 women. Registration offices were asked to provide randomly drawn addresses of women in the age group 20–25 years with primary residence in their municipality. In order to meet the required sample size (see below for sample size calculation) of 1,173 participants, we initially contacted 5,029 women. As response rate was low, a second recruitment wave was started in 2017. *De novo* random sampling of 76 clusters was followed by random sampling of 40 women per cluster and 501 women in the city of Berlin.

An invitation letter was sent to each potential participant including study information and consent form. Study material package was sent to those participants who returned the signed consent form. Study material included a cervicovaginal self-sampling collection kit (Evalyn^®^ Brush, Rovers Medical Devices, Oss, Netherlands) with a detailed description and illustrations for the self-sampling procedure, a paper-based questionnaire or login-data for an online-questionnaire which was developed using the software Voxco (Montreal, Canada). The questionnaire was based on the questionnaire of the 2010–2012 baseline study and was divided into four chapters: (1) general demographic information; (2) information regarding cancer screening and history of HPV-associated dysplastic lesions and cancer; (3) information on sexual behaviour; and (4) information on received HPV vaccination. Invited participants were excluded from the study if they did not sign the consent form, were pregnant, or if no samples were provided. As an incentive, participation in a lottery was offered to all women who completed the study.

To compare participants to non-participants in order to assess possible differences, a short non-responder questionnaire containing questions on age, nationality, education, and pregnancy was sent to all women who did not respond to a postal reminder. The study was conducted according to the German Federal and State Commissioners for Data Protection guidelines and was approved by the Charité—Universitätsmedizin Berlin ethics committee (Reg. No. EA1/094/17) and the German Federal Commissioner for Data Protection. Informed written consent were obtained from all participants.

### HPV testing

Pseudonymised self-collected dry samples were submitted at ambient temperature via postal mail to the RKI. Samples were immediately stored in a fridge at −20°C and were forwarded once per week to Charité HPV laboratory for HPV DNA extraction and testing. Extracted DNA was further stored at −20°C until use. Samples were tested for 18 HR-HPV genotypes (HPV16, 18, 26, 31, 33, 35, 39, 45, 51, 52, 53, 56, 58, 59, 66, 68, 73, 82) and nine LR-HPV genotypes (HPV6, 11, 42, 43, 54, 57, 70, 72, 90) by multiplexed genotyping (MPG) with Luminex readout (16) using BSGP 5+/6+ primer sets. Pseudonymised results were sent back to RKI and re-merged with individual questionnaire data. HPV test results were also forwarded to the participants. Each participant was offered a consultation option by telephone or email. For all participants with a positive HPV result, an appointment with the gynaecologist was recommended and participation in a follow-up study was offered.

### Statistical analysis

For sample size calculation, we assumed that 10% of study participants were infected with HPV16 or HPV18. Based on an assumed minimum VE of 50% and a vaccine coverage of 30%, we aimed to include at least 1,173 participants in the study in order to be able to calculate a significant difference with a power of 80% between vaccinated and non-vaccinated participants in terms of HPV16/18 prevalence at the level of *p* < 0.05. With an expected prevalence of 4%, the presence of at least one of HPV types 31, 33, 35, 52 and 58 could be detected with a precision of 1% in such a sample size. Based on the experience from the baseline survey, we adjusted the expected response rate and therefore increased the gross sample size. Assuming a response rate of about 20%, at least 5,000 invitations had to be sent out initially.

We performed descriptive analyses of study population characteristics and HPV prevalence for individual and grouped genotypes in vaccinated and non-vaccinated women. A participant was defined as vaccinated according to the current STIKO recommendation if her vaccination history met the following criteria: (a) schedule completed before first sexual intercourse, (b) schedule completed before the age of 18 years, and (c) dosing schedule and intervals as recommended by the vaccine manufacturer (17).

HPV prevalence was calculated for all 18 HR-HPV genotypes separately and, in order to assess the effects of the most often used quadrivalent and bivalent vaccines in the study population, for the following groups of HPV genotypes: genotypes HPV16/HPV18 included in the bivalent vaccine (Cervarix^®^) and genotypes HPV6/HPV11/HPV16/HPV18 included in the quadrivalent vaccine (Gardasil^®^). Group-specific prevalence was calculated as the proportion of participants who were DNA-positive for at least one of the HPV-genotypes included in one group. Prevalence was reported as percentage with 95% confidence interval (CI). We performed chi-square tests and Fisher’s exact test for categorical variables, and unpaired Student’s *t*-test for continuous variables. We performed a proportion *t*-test to test for prevalence differences between the results of the baseline study and the present study. We calculated crude and adjusted prevalence ratios (PR) and 95% CIs. To measure the association between HPV positivity and vaccination status and to allow for adjustment, we included all potentially relevant variables (age, nationality, education, smoking, number of sexual partners, immunodeficiency, and cancer screening) and performed Poisson regression with backward selection based on a *p*-value <0.1 to obtain the final model. Prevalence results were further compared descriptively with findings of the baseline study. For a detailed description of the baseline study design, see (9). Based on the adjusted PR we calculated VE with the following equation: VE = (1 – PR) × 100. For calculating VE, we used the definition of being vaccinated as previously described. For all statistical analysis, we used the statistical software STATA^®^, version 17 (StataCorp, TX, United States).

## Results

**Figure 1:**
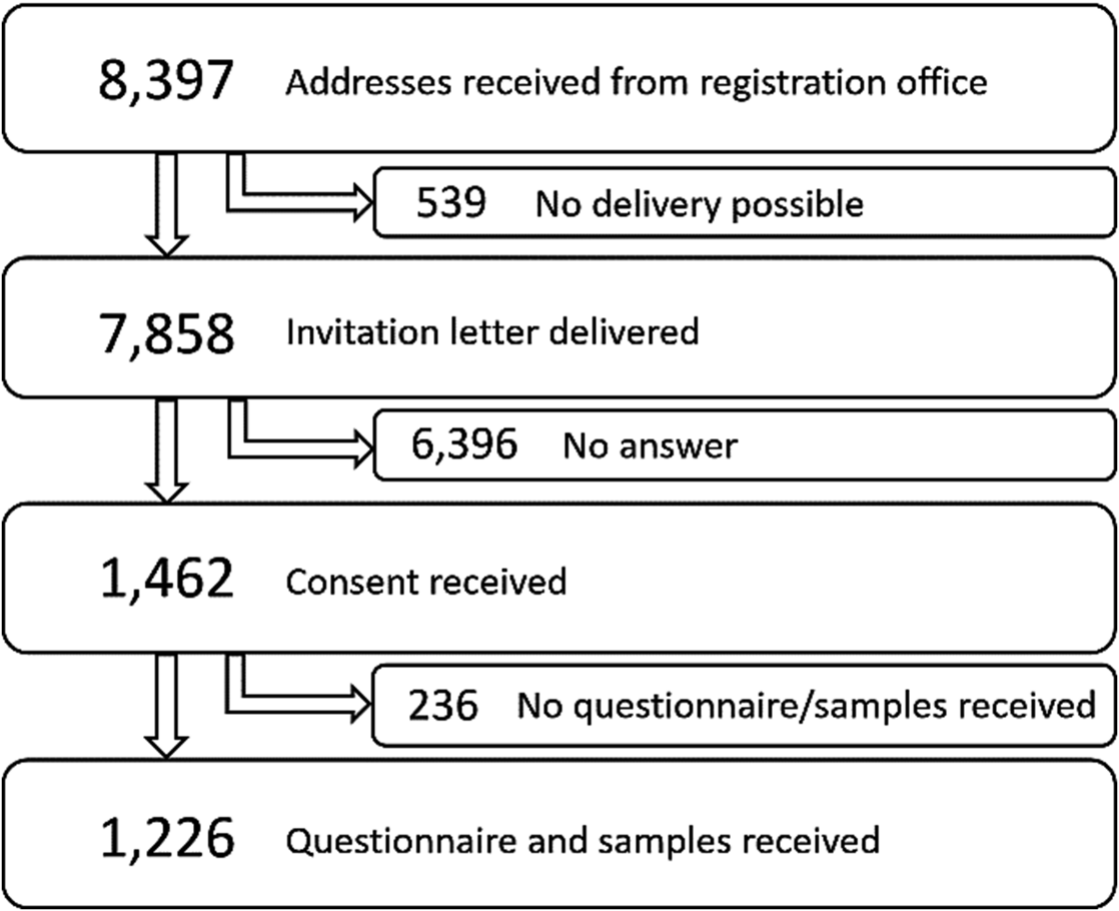
Flow chart of study participant recruitment.

In total 7,858 women were contacted and asked to participate in the study. Of these, 1,226 sent their signed informed consent, cervicovaginal sample and a completed questionnaire to the RKI (response rate 15.6%) (Figure 1). A non-responder questionnaire was completed by 607 of the women who did not participate in the study. Of these, 47 (7.7%) were pregnant. The remaining 556 non-participants were similar to participants regarding age and region of residence but differed in nationality and educational background (8.5% vs. 2.7% non-German citizenship and 4.9% vs. 2.4% with low level of education in non-participants and participants, respectively).

Demographic characteristics of participants (*n* = 1,226) are provided in Table 1. Mean age of participants was 23 years. With the exception of the 20 year-olds (*n* = 128), the study included about 200 participants per predefined age group. Although recruitment targeted only 20–25 year-olds, few women (*n* = 16) who turned 26 between recruitment and participation were included in the study. Participants came from all regions of Germany and were evenly distributed between rural and urban regions as well as eastern and western parts of Germany. The majority (95%; *n* = 1,170) of participants had a German citizenship. In total, 2.4% (*n* = 29) of participants had a low education status (based on secondary school qualifications).

**Table 2:** Sexual behaviour and vaccine uptake of the study participants stratified by HPV16/HPV18 positivity.

	Total	HPV16/HPV18-negative	HPV16/HPV18-positive	*p*-value
	*N* = 1,226	*N* = 1,132	*N* = 94	
**Current relationship**				0.046
No	354 (28.9%)	316 (27.9%)	38 (40.4%)	
Yes	846 (69.0%)	793 (70.1%)	53 (56.4%)	
Not specified	2 (0.2%)	2 (0.2%)	0 (0.0%)	
Missing	24 (2.0%)	21 (1.9%)	3 (3.2%)	
**Lifetime number sexual partners** (mean & IQR)	4 (1–7)	3 (1–7)	9 (5–15)	**<0.001**
**Lifetime number male sexual partners** (mean & IQR)	4 (2–7)	3 (2–6)	8 (4–14)	**<0.001**
**Lifetime number female sexual partners** (mean & IQR)	0 (0–0)	0 (0–0)	0 (0–1)	**0.026**
**Number sexual partners 12 months**				**<0.001**
0	238 (19.4%)	231 (20.4%)	7 (7.4%)	
1	667 (54.4%)	638 (56.4%)	29 (30.9%)	
2	144 (11.7%)	127 (11.2%)	17 (18.1%)	
3–5	119 (9.7%)	93 (8.2%)	26 (27.7%)	
>5	30 (2.4%)	18 (1.6%)	12 (12.8%)	
Not specified	4 (0.3%)	4 (0.4%)	0 (0.0%)	
Missing	24 (2.0%)	21 (1.9%)	3 (3.2%)	
**Age sexual debut**				0.13
No sexual intercourse yet	60 (4.9%)	60 (5.3%)	0 (0.0%)	
<13 years	6 (0.5%)	6 (0.5%)	0 (0.0%)	
13 years	49 (4.0%)	44 (3.9%)	5 (5.3%)	
14 years	139 (11.3%)	122 (10.8%)	17 (18.1%)	
15 Years	178 (14.5%)	168 (14.8%)	10 (10.6%)	
16 years	247 (20.1%)	225 (19.9%)	22 (23.4%)	
17 years	179 (14.6%)	166 (14.7%)	13 (13.8%)	
>17 years	342 (27.9%)	318 (28.1%)	24 (25.5%)	
Missing	26 (2.1%)	23 (2.0%)	3 (3.2%)	
**Condom use in current relationship**				**0.013**
No	523 (42.7%)	469 (41.4%)	54 (57.4%)	
Yes	629 (51.3%)	595 (52.6%)	34 (36.2%)	
Not specified	50 (4.1%)	47 (4.2%)	3 (3.2%)	
Missing	24 (2.0%)	21 (1.9%)	3 (3.2%)	
**Condom use with new partners**				0.32
No	87 (7.1%)	82 (7.2%)	5 (5.3%)	
Yes	981 (80.0%)	901 (79.6%)	80 (85.1%)	
Not specified	134 (10.9%)	128 (11.3%)	6 (6.4%)	
Missing	24 (2.0%)	21 (1.9%)	3 (3.2%)	
**Oral contraceptives**				0.45
No	140 (11.4%)	132 (11.7%)	8 (8.5%)	
Yes	1,050 (85.6%)	967 (85.4%)	83 (88.3%)	
Not specified	12 (1.0%)	12 (1.1%)	0 (0.0%)	
Missing	24 (2.0%)	21 (1.9%)	3 (3.2%)	
**HPV vaccination (at least one dose)**				**0.037**
No	377 (30.8%)	338 (29.9%)	39 (41.5%)	
Yes	757 (61.7%)	712 (62.9%)	45 (47.9%)	
I don’t know	60 (4.9%)	53 (4.7%)	7 (7.4%)	
Missing	32 (2.6%)	29 (2.6%)	3 (3.2%)	
**Cervarix**				0.69
No	1,163 (94.9%)	1,073 (94.8%)	90 (95.7%)	
Yes	63 (5.1%)	59 (5.2%)	4 (4.3%)	
**Gardasil**				**0.004**
No	594 (48.5%)	535 (47.3%)	59 (62.8%)	
Yes	632 (51.5%)	597 (52.7%)	35 (37.2%)	
**Gardasil9**				0.39
No	1,217 (99.3%)	1,123 (99.2%)	94 (100.0%)	
Yes	9 (0.7%)	9 (0.8%)	0 (0.0%)	
**Vaccination (recommended scheme)**				**0.022**
No	377 (30.8%)	338 (29.9%)	39 (41.5%)	
Yes	348 (28.4%)	331 (29.2%)	17 (18.1%)	
Missing	501 (40.9%)	463 (40.9%)	38 (40.4%)	

Statistically significant *p*-values in bold.

Of the participating women, 20% (*n* = 245) were active and 8% (*n* = 101) former smokers. Thirty women (2.4%) had some form of immunodeficiency and were taking respective medication. Thirty-four percent (*n* = 420) of participants had never previously participated in cervical (pre-)cancer screening. Five percent (*n* = 55) of the participants were previously diagnosed with a cervical lesion and 15 (1.2%) reported having received genital warts treatment in the past.

Demographic characteristics were additionally stratified by HPV16/18 positivity (Table 1). While 1,132 participants were negative, the remaining 94 women were DNA positive for at least one of the two HPV types 16 and 18. We did not find statistically significant differences in demographics between HPV16/18 positive and HPV16/18 negative participants. However, the proportion of participants with a history of cervical lesion diagnosis was significantly higher in those who were HPV16/18 positive (*p* < 0.001).

**Table 1:** Demographic characteristics of the study participants stratified by HPV16/HPV18 positivity.

	Total	HPV16/HPV18-negative	HPV16/HPV18-positive	*p*-value
	*N* = 1,226	*N* = 1,132	*N* = 94	
**Age** (mean & IQR)	23 (21–24)	23 (21–24)	23 (21–24)	0.26
**Region of residence**				0.55
Berlin	94 (7.7%)	85 (7.5%)	9 (9.6%)	
East—rural	243 (19.8%)	228 (20.1%)	15 (16.0%)	
West—rural	290 (23.7%)	272 (24.0%)	18 (19.1%)	
East—urban	349 (28.5%)	318 (28.1%)	31 (33.0%)	
West—urban	250 (20.4%)	229 (20.2%)	21 (22.3%)	
**German citizenship**				0.62
No	32 (2.6%)	29 (2.6%)	3 (3.2%)	
Yes	1,170 (95.4%)	1,082 (95.6%)	88 (93.6%)	
Missing	24 (2.0%)	21 (1.9%)	3 (3.2%)	
**Education**				0.074
Low education	29 (2.4%)	27 (2.4%)	2 (2.1%)	
Medium education	202 (16.5%)	179 (15.8%)	23 (24.5%)	
High education	967 (78.9%)	902 (79.7%)	65 (69.1%)	
Missing	28 (2.3%)	24 (2.1%)	4 (4.3%)	
**Income**				0.070
<1,000 EUR	413 (33.7%)	374 (33.0%)	39 (41.5%)	
1,000–2,00 EUR	346 (28.2%)	314 (27.7%)	32 (34.0%)	
2,000–3,000 EUR	231 (18.8%)	220 (19.4%)	11 (11.7%)	
>3,000 EUR	200 (16.3%)	191 (16.9%)	9 (9.6%)	
Missing	36 (2.9%)	33 (2.9%)	3 (3.2%)	
**Smoking**				0.30
No	854 (69.7%)	794 (70.1%)	60 (63.8%)	
Yes	245 (20.0%)	220 (19.4%)	25 (26.6%)	
Not anymore	101 (8.2%)	95 (8.4%)	6 (6.4%)	
Not specified	26 (2.1%)	23 (2.0%)	3 (3.2%)	
**Immunodeficiency**				0.17
No	1,160 (94.6%)	1,075 (95.0%)	85 (90.4%)	
Yes	30 (2.4%)	26 (2.3%)	4 (4.3%)	
Missing	36 (2.9%)	31 (2.7%)	5 (5.3%)	
**Cervical cancer screening**				0.71
Never	420 (34.3%)	392 (34.6%)	28 (29.8%)	
Yes, once	200 (16.3%)	182 (16.1%)	18 (19.1%)	
Yes, regularly	411 (33.5%)	381 (33.7%)	30 (31.9%)	
I don’t know	170 (13.9%)	155 (13.7%)	15 (16.0%)	
Missing	25 (2.0%)	22 (1.9%)	3 (3.2%)	
**Cervical lesions**				**<0.001**
No	1,097 (91.3%)	1,022 (92.0%)	75 (82.4%)	
Yes	55 (4.6%)	42 (3.8%)	13 (14.3%)	
I don’t know	46 (3.8%)	43 (3.9%)	3 (3.3%)	
Missing	4 (0.3%)	4 (0.4%)	0 (0.0%)	
**Treatment genital warts**				0.54
No	1,179 (96.2%)	1,090 (96.3%)	89 (94.7%)	
Yes	15 (1.2%)	13 (1.1%)	2 (2.1%)	
I don’t know	8 (0.7%)	8 (0.7%)	0 (0.0%)	
Not specified	24 (2.0%)	21 (1.9%)	3 (3.2%)	
Missing	1,179 (96.2%)	1,090 (96.3%)	89 (94.7%)	

IQR, interquartile range. Statistically significant *p*-values in bold.

We further analysed participants according to sexual behaviour and vaccine uptake (Table 2). In total, 69% reported to currently being in a relationship. The mean lifetime number of sexual partners was four (interquartile range: 1–7). Within the previous 12 months, the majority (54%) of participants had one sexual partner and 19% had no sexual partner. Five percent of the women never had sexual intercourse before, while 16% had their sexual debut at the age of 14 years or earlier. A vast majority (86%) of women used oral contraceptives (Table 2).

Regarding vaccination history, 62% had received at least one dose of either Cervarix (*n* = 63), Gardasil (*n* = 632) or Gardasil9 (*n* = 9). In total, 348 (28%) of the women were vaccinated according to current STIKO recommendations. Nearly half (48%) of the participants who provided information on their age at the time of the first vaccine dose were vaccinated when they were older than 14 years (Supplementary Figure S1). Of the 356 women who were vaccinated before age 15, around 10% (*n* = 36) had their first vaccine dose in the same year as their sexual debut or after their sexual debut.

We found statistically significant differences between HPV16/18 positive and HPV16/18 negative participants regarding sexual behaviour and vaccine uptake. HPV16/HPV18-positive participants were less likely to be living in a current relationship (58% versus 71%; *p*-value: 0.026), had a higher lifetime number of sexual partners (mean number: 9 versus 3; *p*-value: <0.001) as well as a higher number of sexual partners in the past 12 months (>1 sexual partner: 61% versus 21%; *p*-value: <0.001). Moreover, they reported using condoms less often during sexual intercourse in relationships (37% versus 54%; *p*-value: 0.007). Regarding vaccination history, HPV16/18 positives were less likely to have an HPV vaccination history (no HPV vaccine dose: 43% versus 31%; *p*-value: 0.015).

### Prevalence of HPV genotypes by vaccination status

Prevalence of HPV genotypes is reported in Supplementary Figures S2, S3.

The prevalence of the vaccine-covered HPV genotypes ranged from 0% (95% CI 0.0–0.5) for HPV11 to 7.6% (95% CI 6.2–9.2) for HPV52 (Table 3). HPV prevalence for HPV16 and HPV18 was 7.0% (95% CI 5.6–8.6) and 0.8% (95% CI 0.4–1.5), respectively. The LR types HPV6 and HPV11 were rare with only five (0.4%; 0.1–0.9) and one (0%; 95% CI 0.0–0.5) positive tests among the 1,226 participants. For all vaccine-covered and other HR types except of HPV58, prevalence was lower among vaccinated compared to unvaccinated participants. For HPV16/18, prevalence among vaccinated was 4.9% (95% CI 2.9–7.7), compared to 10.3% (95% CI 7.5–13.9) among the unvaccinated women (*p*-value <0.01). Analyzing the HPV type-specific prevalence by age of first vaccination dose before and after the participants’ 15th birthday showed no significant differences (data not shown).

**Table 3:** HPV prevalence by vaccination status.

HPV types	Total (*N* = 1,226)		Unvaccinated participants (*N* = 377)		Vaccinated participants (*N* = 348)		*p*-value
	*N*	% (95% CI)	*N*	% (95% CI)	*N*	% (95% CI)	
**Genotypes** ^b^ **covered by the quadrivalent vaccine**							
HPV6	5	0.4 (0.1–0.9)	4	1.1 (0.3–2.7)	0	0 (0–1.1)	**0.0450**
HPV11	1	0 (0.0–0.5)	1	0.3 (0–1.5)	0	0 (0–1.1)	0.3170
HPV6/11^a^	6	1.3 (0.4–3.1)	5	1.3 (0.4–3)	0	0 (0–1.1)	**0.0249**
HPV16	86	7.0 (5.6–8.6)	31	8.2 (5.7–11.5)	17	4.9 (2.9–7.7)	0.0683
HPV18	10	0.8 (0.4–1.5)	10	2.7 (1.3–4.8)	0	0 (0–1.1)	**0.0015**
HPV16/18^a^	94	7.7 (6.2–9.3)	39	10.3 (7.5–13.9)	17	4.9 (2.9–7.7)	**0.0053**
HPV6/11/16/18^a^	97	7.9 (6.5–9.6)	42	11.1 (8.1–14.7)	17	4.9 (2.9–7.7)	**0.0018**
**Additional HR genotypes** ^b^ **covered by the nine-valent vaccine**							
HPV31	18	1.5 (0.9–2.3)	10	2.7 (1.3–4.8)	2	0.6 (0.1–2.1)	**0.0246**
HPV33	18	1.5 (0.9–2.3)	8	2.1 (0.9–4.1)	3	0.9 (0.2–2.5)	0.1586
HPV45	31	2.5 (1.7–3.6)	11	2.9 (1.5–5.2)	7	2.0 (0.8–4.1)	0.4300
HPV52	93	7.6 (6.2–9.2)	33	8.8 (6.1–12.1)	21	6.0 (3.8–9.1)	0.1608
HPV58	12	1.0 (0.5–1.7)	1	0.3 (0–1.5)	5	1.4 (0.5–3.3)	0.0905

a HPV6/11, HPV6 and/or HPV11; HPV16/18, HPV16 and/or HPV18; HPV6/11/16/18, HPV6 and/or HPV11 and/or HPV16 and/or HPV18.

b At the time of our study, vaccine-covered genotypes were HPV6, 11, 16, and 18 as only a marginal proportion of participants were vaccinated with Gardasil9 including HPV31, 33, 45, 52, and 58.Statistically significant *p*-values in bold.

### Vaccine effectiveness and impact on HPV prevalence

Confounder-adjusted VE was 46.4% (95% CI 4.2–70.1) against HPV16/18 infection (Table 4). VE against infection with at least one of the four HPV genotypes covered by the quadrivalent vaccine, i.e., HPV6/11/16/18 was 49.1% (95% CI 8.2–71.8).

**Table 4:** Crude and adjusted prevalence ratio and vaccine effectiveness for vaccine-covered HPV groups.

HPV genotypes	Crude PR (95% CI)	*p*-value	VE (95% CI) (for crude PR)	Adjusted PR (95% CI)	*p*-value	VE (95% CI) (for adjusted PR)
HPV16, 18	0.5 (0.3–0.8)	**0.010**	52.8 (16.5–73.3)	0.5 (0.3–1.0)	**0.035**	46.4 (4.2–70.1)
HPV6/11/16/18^a^	0.4 (0.3–0.8)	**0.006**	55.1 (20.2–74.8)	0.5 (0.3–0.9)	**0.025**	49.1 (8.2–71.8)

PR, prevalence ratio; VE, vaccine effectiveness.

a Calculation of PR and VE for HPV6, 11, 16, 18 only for participants with Gardasil/Gardasil9 vaccination.Statistically significant *p*-values in bold.

Compared with the baseline study results, overall HPV16/18 prevalence significantly decreased from 20.2% (95% CI 17.5–23.2) in 2010–2012 to 7.7% (95% CI 6.2–9.3) in 2017/2018 in the study populations (9). We further compared the prevalence of vaccine-covered HPV genotypes among the non-vaccinated participants in the present study with those calculated in the baseline study (Table 5). While HPV6 prevalence remained relatively stable, HPV11, HPV16, HPV18 and HPV58 prevalence decreased by 50% between 2010–2012 and 2017/2018. However, this was only statistically significant for HPV16 (*p*-value <0.0001). For the vaccine-covered genotypes HPV16/18, prevalence dropped from previously 22.5% (95% CI 19.0–26.3) to 10.3% (95% CI 7.5–13.9; *p* < 0.0001). Interestingly, we observed an increase in HR genotypes that are not included in the bivalent and quadrivalent HPV-vaccines (HPV31, HPV33, HPV45 and HPV52) when comparing the results of the baseline study with the present study results.

**Table 5:** HPV prevalence for vaccine-covered and additional high-risk (HR) HPV genotypes among non-vaccinated participants in the baseline study (2010–2012) and present study (2017/2018).

HPV genotypes	2010–2012 (*N* = 512)		2017/2018 (*N* = 377)		*p*-value^a^
	*N*	% (95% CI)	*N*	% (95% CI)	
Genotypes^b^ covered by the quadrivalent vaccine					
HPV6	5	1.0 (0.3–2.3)	4	1.1 (0.3–2.7)	0.9011
HPV11	4	0.8 (0.1–2.0)	1	0.3 (0–1.5)	0.3093
HPV16	100	19.5 (16.2–23.2)	31	8.2 (5.7–11.5)	**<0.0001**
HPV18	26	5.1 (3.3–7.3)	10	2.7 (1.3–4.8)	0.0698
HPV16/18	115	22.5 (19.0–26.3)	39	10.3 (7.5–13.9)	**<0.0001**
**Additional HR genotypes** ^b^ **covered by the nine-valent vaccine**					
HPV31	2	0.4 (0.1–1.4)	10	2.7 (1.3–4.8)	**0.0039**
HPV33	3	0.6 (0.1–1.7)	8	2.1 (0.9–4.1)	**0.0406**
HPV45	1	0.2 (0.0–1.0)	11	2.9 (1.5–5.2)	**0.0005**
HPV52	11	2.2 (1.1–3.8)	33	8.8 (6.1–12.1)	**<0.0001**
HPV58	5	1.0 (0.3–2.3)	1	0.3 (0–1.5)	0.2005

a *p*-value for test of proportion difference.

b At the time of our study, vaccine-covered genotypes were HPV6, 11, 16, and 18 as only a marginal proportion of participants were vaccinated with Gardasil9 including HPV31, 33, 45, 52, and 58.Statistically significant *p*-values in bold.

## Discussion

This study reports population-based data on HPV genotype-specific prevalence based on self-sampling among young women 10 years after initiation of routine HPV vaccination in Germany. With these analyses, we were able to evaluate the impact of the vaccination program in terms of reduction of HPV infections at population level 10 years after its initiation and to estimate vaccine effectiveness of HPV vaccines.

### Vaccination history/vaccine uptake

HPV vaccine coverage in adults is not routinely measured and reported in Germany, but data on coverage in adolescents are available: In 2008, shortly after endorsement of the HPV-vaccination recommendation, HPV vaccine uptake in 12–17 years-old girls in Germany was around 32% (18). In 2011, health insurance claims data from Germany calculated a vaccine uptake of 27% among 15 years-olds (14).

In our investigated cohort of 20–25 year-old women from 2017/2018, vaccine uptake was substantially higher compared to the above described data on vaccine uptake in the respective birth cohorts. The majority (63%) of the participating women aged 20–25 had initiated and 55% completed an HPV vaccine series. In terms of comparison of these studies, it is important to mention that we investigated an older age group compared with the age groups of the previously described studies. Besides this circumstance, the possible bias of voluntary responses (vaccinated individuals are more likely to respond to an invitation to the study than unvaccinated individuals) could also be the cause of this difference in vaccine uptake. Our results—gained from a study population with a higher vaccine coverage than the general population—might overestimate the reduction of HPV prevalence in the population and, thereby, the estimated effect of the vaccination program. However, the higher vaccine coverage in our study population has neither an effect on the VE estimates nor on the results regarding the reduction of HPV prevalence since 2010–2012 in vaccinated women as well as in unvaccinated women.

When our study population was eligible for vaccination, the vaccination program targeted only 12–17 years-old girls. Earlier vaccination (9–14 years of age allowing for catch-up vaccination until 18 years and before sexual debut) was introduced in Germany in 2014 as a recommendation to prevent HPV infections more effectively. Still, 28% of the women in our study had been vaccinated according to the current recommendation, so that we were able to evaluate the difference in prevalence among vaccinated and non-vaccinated participants as well as the VE according to the current vaccination scheme.

A high number of women started their vaccination series after their 15th birthday (48%) or initiated the vaccination series before turning 15 years but after sexual debut or at least in the same year (10%). Since the time of vaccination of this study population, recommended age limits were lowered by STIKO in Germany. Retrospectively, these findings strongly support the decision of STIKO in 2014 to lower the recommended HPV vaccination age from 12–17 years to 9–14 years ensuring a timely vaccination before sexual debut. Still, it has to be emphasized that vaccination should be completed before sexual debut irrespective of age as >16% of our study population had their sexual debut prior to their 15th birthday.

### HPV prevalence and comparison to results of the baseline study

Vaccine-covered HPV genotypes were rare in our study cohort, and overall the majority of HPV genotypes had a prevalence of ≤2.5%. Only HPV16 and HPV52 had a higher prevalence of 7.0 and 7.6%, respectively. With an overall HPV16/18 prevalence of 7.7%, the prevalence was approximately 50% lower in vaccinated (4.9%) than in non-vaccinated women (10.3%) showing the direct effect of vaccination. With the exception of HPV58, all HPV genotypes were less prevalent in the group of vaccinated participants. We observed an increase in prevalence of non-vaccine HPV genotypes in HPV vaccinated women as compared to unvaccinated women and the baseline study. A possible explanation could be the differences of the induced immune responses during spontaneous resolution of an HPV infection, leading to a broad poliantigenic response also against the more conserved viral E proteins, while L1-based virus like particle (VLP) vaccination induces exclusively L1 genotype-specific neutralizing antibodies and L1-specific T cell responses in the absence of other HPV antigens. These more diverse immune reactivities against more conserved early proteins will comprise cross-protective immunity against related HPV genotypes that in turn will not be able to establish an infection. However, after L1 VLP vaccination this cross-protection is not present and not protecting against related HPV genotypes that now can infect and their prevalence seems to increase. Despite an increased prevalence the carcinogenicity of the individual HPV genotypes is not enhanced.

Among women aged 20–25 years, the HPV genotype prevalence covered by both the bivalent and quadrivalent vaccine, i.e., HPV16 and HPV18, decreased from 20% in 2012 (baseline study) to 8% in 2017/2018. Prevalence of HPV16/18 among non-vaccinated women in our present study was significantly lower compared to non-vaccinated women from the baseline study (10% vs. 23%, *p* < 0.0001). This significant reduction in HPV16 and HPV18 prevalence among unvaccinated women can be an explained by herd protection effects conferred by the implemented HPV vaccination program, as also reported from other populations (19, 20).

Prevalence of HPV types 31, 33, 45, and 52 among unvaccinated women was significantly higher in our study compared to the results of the baseline study. Whether this may indicate a vaccine-induced HPV genotype replacement is unclear (21). However, it is well known that the bivalent vaccine leads to higher cross-protection against prevalent infection with types 31, 33 and 45 in comparison to the quadrivalent vaccine (20, 22). The vast majority of vaccinated women in our study were vaccinated with the quadrivalent vaccine (90%) and only 9% with the bivalent vaccine. As the nine-valent vaccine was not introduced before 2016, our study population did not benefit from the nine-valent vaccine and was only marginally cross-protected since the bivalent vaccine was rarely used.

### Vaccine effectiveness

On first glance, the VE estimate of about 50% obtained in our study appears to be relatively low, compared to the high efficacy estimates of 80%–90% against incident and persistent infections obtained in randomized controlled trials (RCT) (for systematic review of the trial data, see (12)). However, we did not conduct an RCT but an observational study that reflects real life conditions. Data on HPV vaccination were self-reported by the participating women. Neither we could verify whether they were actually HPV naïve before vaccination or had not yet had sexual intercourse. At least four arguments have to be considered when interpreting our data: (1) because HPV infection was only measured at a single point in time in our study (based on the study design), we could not distinguish between incident and persistent infection or even transient HPV DNA positivity. This might have introduced imprecision into our estimate. (2) Although being statistically significant, our VE estimates had very wide 95% CIs. The upper limit of the 95% CI against HPV16/18 infection indicates that the “true” value could also be around 70%. (3) Some studies, using the same methodological approach (vagino-cervical self-sampling) in similar age groups of women, have reported higher VE point estimates: Batmunkh et al. (23) observed a 1-dose effectiveness of 92% in young women in Mongolia, and Hoes et al. (24) reported a VE of 84% against incident infections after vaccination with Cervarix. However, the latter study [Hoes et al. (24)] reported a very wide 95% CI, which overlaps with the 95% CI of our study. (4) At least one further study [Guo et al. (25)], using the US-American NHANES study dataset, observed a VE of the same magnitude (46%) as our study (25). This result was recently confirmed in an updated analysis of the same study population (26). Overall, the published VE estimates against HPV infection from self-sampling data show some degree of heterogeneity of unknown origin which should be investigated in further studies.

### Strengths and limitations

Our study has several strengths. It used a nation-wide sampling frame to recruit participants with diverse socio-economic backgrounds from the resident population in Germany. Applying a well-established test kit and self-sampling method, we were able to obtain HPV prevalence that is unlikely to be confounded by health-care seeking behaviour. We could use detailed data on factors like pre-existing co-morbidities, socio-economic status, sexual behaviour and former vaccination to perform our analyses. Even though the self-sampling material used in the baseline study was different to the one used in the present study (cervicovaginal lavage versus dry brush sampling), both studies had a similar study design and we were therefore able to compare HPV genotype-specific prevalence data in 2017/18 with those in 2010–12 and to demonstrate the extent of the impact of the German HPV vaccination strategy on population level including indirect effects such as herd immunity.

However, our study also has limitations. The present study had a low response rate of only 15.6% in spite of two recruitment waves and the use of incentives. Non-responders were more often of non-German citizenship and of lower education background than participants. Considering the intense cost- and time-consuming effort in reaching the sample size minimum, this study design seems to be not efficient enough for estimating population-based prevalence data of HPV in Germany. Moreover, the target population of our study is generally considered to be one of the harder to reach populations at least in Germany. Additionally, we may have a common response bias regarding our questions targeting sexual behaviour (e.g., sexual debut). Like other population-based cross-sectional studies, we may additionally have a healthy population bias, which leads to a potentially higher number of women who are interested in healthy behaviour and prevention of HPV-infections. Furthermore, HPV infection at only one time point might not be the relevant outcome to estimate vaccine effectiveness and future studies in Germany should focus on persistent infections or even more informative endpoints than genotype-specific prevalent infection.

### Summary

In summary, the prevalence of vaccine-covered HPV genotypes among the 20–25 year-old participants in Germany was low and decreased compared with HPV prevalence estimated shortly after the introduction of the HPV vaccination program. In comparison to unvaccinated women, HPV prevalence of almost all vaccine-covered genotypes was strongly reduced in vaccinated participants. Interestingly, a significant decrease of HPV16 and HPV18 was even observed in unvaccinated participants, compared to baseline study results, indicating indirect protection of unvaccinated women through herd immunity effects. Estimated VE against HPV16/18 and HPV6/11/16/18 was rather low in our study, assuming that the study design in combination with the endpoint selection of (mainly transient) HPV DNA positivity is not an effective and appropriate study design for defining HPV VE. This study presents additional data showing that since the introduction of HPV vaccination in Germany, HPV prevalence of the most relevant HPV types HPV16 and HPV18 further decreased in the German female population compared to prevalence data from the baseline study.

In many participants, vaccination was not received as recommended before sexual debut. Therefore, there is an urgent need to raise awareness to physicians and the public that a large group of girls is not vaccinated, and even if they are, they are not vaccinated according to STIKO recommendations, and that these girls are not receiving the full potential of vaccine protection against HPV infections and associated cervical dysplasia. To ensure that girls are vaccinated before sexual debut and that the series of vaccinations is completed before their 15th birthday, HPV vaccination should be given starting at age 9 years when girls and boys are still being managed in pediatric care.

## Data availability statement

The raw data supporting the conclusions of this article will be made available by the authors, without undue reservation.

## Ethics statement

The studies involving human participants were reviewed and approved by Charité—Universitätsmedizin Berlin Ethics Committee (Reg. No. EA1/094/17). The patients/participants provided their written informed consent to participate in this study.

## Author contributions

MW-P, TH, OW, and AK designed the study. AL, AT, VS, and AM conducted the study, prepared the data base, and performed statistical analyses. AK and ST performed laboratory analyses. MS developed the sampling strategy. MW-P, OW, and TH supervised the study conduct and data analysis. AL, AT, and VS developed the first manuscript draft. AL, VS, AT, MW-P, AM, ST, MS, AK, OW, and TH contributed to the interpretation of the data and provided important intellectual content to the manuscript. All authors contributed to the article and approved the submitted version.
