# Supplementary material for: Human papillomavirus prevalence and vaccine effectiveness in young women in Germany, 2017/2018: results from a nationwide study
Source: Front Public Health. 2023 Aug 31;11:1204101. doi: 10.3389/fpubh.2023.1204101 (PMC10501861; doi:10.3389/fpubh.2023.1204101)
Supplement: Supplementary file 3 [file Table_3.docx]

Supplementary Material

**Human Papillomavirus (HPV) prevalence and vaccine effectiveness in young women in Germany, 2017/2018: results from a nationwide study**

**Anna Loenenbach, Viktoria Schönfeld, Anja Takla, Miriam Wiese-Posselt, Adine Marquis, Sarah Thies, Matthias Sand, Andreas M. Kaufmann, Ole Wichmann, Thomas Harder**

*** Correspondence: Corresponding Author: taklaa@rki.de**


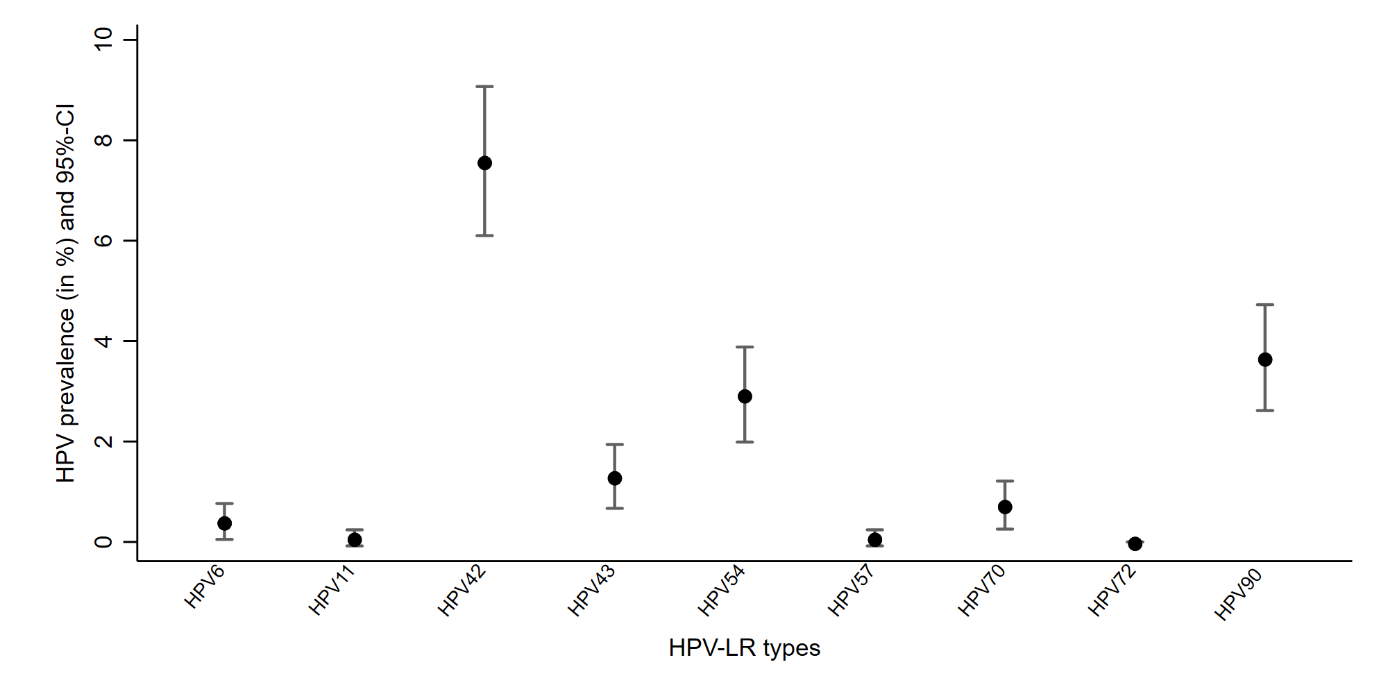


Supplementary Figure 3. HPV prevalence and 95% confidence intervals (CI) of 9 low risk (LR) types; HPV prevalence study (n=1,226).
